# Reevaluating the Role of Bronchoscopy Prior to Bronchial Artery Embolization in Nonintubated Patients With Hemoptysis Due to Bronchiectasis and Chronic Pulmonary Infection

**DOI:** 10.1016/j.chpulm.2024.100128

**Published:** 2024-12-10

**Authors:** Takashi Nishihara, Hideo Ishikawa, Kazunari Tsuyuguchi, Shoichi Fukuda, Hiromitsu Sumikawa

**Affiliations:** a Department of Internal Medicine, National Hospital Organization Kinki Chuo Chest Medical Center, Japan; b Department of Infectious Diseases, Clinical Research Center, National Hospital Organization Kinki Chuo Chest Medical Center, Japan; c Department of Radiology, National Hospital Organization Kinki Chuo Chest Medical Center, Japan; d Hemoptysis and Pulmonary-Circulation Center, Eishinkai Kishiwada Rehabilitation Hospital, Japan

**Keywords:** bronchiectasis, bronchoscopy, embolization, hemoptysis, respiratory tract infections

## Abstract

**Background:**

When performing bronchial artery embolization (BAE), identifying the side of bleeding and thereby deciding the side of embolization is crucial for an effective and safe procedure. However, there is little evidence regarding the utility of bronchoscopy for determining the side of embolization prior to BAE in nonintubated patients with hemoptysis admitted to general wards.

**Research Question:**

Is bronchoscopy necessary prior to BAE in nonintubated patients with hemoptysis following bronchiectasis and chronic pulmonary infection?

**Study Design and Methods:**

Data from 93 consecutive nonintubated general ward patients with bronchiectasis and chronic pulmonary infection (nontuberculous mycobacteriosis, aspergillosis, and TB) who underwent de novo BAE from September 2017 to August 2023 were retrospectively reviewed. The contribution of bronchoscopy in deciding the side of embolization was evaluated.

**Results:**

All patients underwent CT imaging and 27 also underwent bronchoscopy. Bronchoscopy identified the sides of bleeding in 9 patients, but these sides could be correctly estimated in 8 of them from the CT information alone. Bronchoscopy did not reveal the side of bleeding in 18 patients, whose sides of embolization were decided using CT imaging and angiographic information. Of 66 patients without bronchoscopy, the sides of embolization were decided in 63 patients using CT imaging and angiographic information, but the priority of the embolization side could not be decided in the remaining 3 patients. Overall, 96% (89 of 93) of patients did not require bronchoscopy as part of their embolization plan. The 90-day overall survival and hemoptysis-free survival rates were 98.9% (95% CI, 92.5-99.8) and 92.3% (95% CI, 84.6-96.3), respectively.

**Interpretation:**

This study showed that bronchoscopy contributed little to the planning of BAE in nonintubated patients with hemoptysis following bronchiectasis and chronic pulmonary infection. Our findings do not support the routine use of bronchoscopy prior to BAE in this population.

**Take-Home Points**

**Study Question:** Is bronchoscopy necessary prior to bronchial artery embolization (BAE) in nonintubated patients with hemoptysis caused by bronchiectasis and chronic pulmonary infection?

**Results:** This study showed that bronchoscopy contributed little to the planning of BAE in nonintubated patients with hemoptysis caused by bronchiectasis and chronic pulmonary infection.

**Interpretation:** Our findings do not support the routine use of bronchoscopy prior to BAE in this population.

Flexible bronchoscopy for hemoptysis is used for distinguishing the site of bleeding, for etiologic diagnosis, and for therapeutic purposes (eg, intubation, bronchial blocker placement, hemostatic techniques). However, the clinical utility of bronchoscopy varies according to the phase, severity, and etiology.^1^, ^2^, ^3^, ^4^, ^5^ Bronchial artery embolization (BAE) is one of the optimal treatments for both massive/life-threatening and mild to moderate hemoptysis; when performing BAE, lateralization is crucial for a safe and effective procedure.^4^^,^^6^^,^^7^ However, lateralization is often problematic in patients with hemoptysis due to bronchiectasis and chronic pulmonary infection (eg, nontuberculous mycobacteriosis, aspergillosis, TB), the leading causes of hemoptysis and common indications for BAE,^8^, ^9^, ^10^ because these patients usually have bilateral lung involvement and concomitant multiple nonbronchial systemic arteries.^11^

Several studies on massive hemoptysis, including in ICU patients, reported that CT imaging revealed the site of bleeding in 70% to 89% of cases, questioning the utility of bronchoscopy prior to BAE.^7^^,^^12^ Furthermore, there is little evidence regarding the efficacy and safety of bronchoscopy for lateralization prior to BAE in nonintubated patients with hemoptysis admitted to general wards, although the risks of bronchoscopy are of greater concern (eg, airway compromise due to sedation, delay in definitive treatment, hypoxemia, high cost).^1^^,^^6^^,^^7^^,^^9^ The aim of the current study therefore was to evaluate the role of bronchoscopy in the planning of BAE in nonintubated patients with hemoptysis caused by bronchiectasis and chronic pulmonary infection.

## Study Design and Methods

### Study Participants

This single-center, retrospective, observational study was conducted over 6 years from September 2017 to August 2023. This study was approved by the ethics committee of the National Hospital Organization Kinki Chuo Chest Medical Center (Institutional Review Board number 2023-60) and complied with the tenets of the Declaration of Helsinki.

The opt-out method was used to obtain permission to use medical records of patients.

During the study period, 154 consecutive patients with hemoptysis underwent de novo BAE. Of these, those with bronchiectasis or chronic pulmonary infections were eligible to be included in the analysis. The exclusion criteria were patients who: (1) required intubation or mechanical ventilation; (2) had blood-streaked sputum; (3) did not have hemoptysis; and (4) had refused treatment prior to completion of all BAE sessions. Hemoptysis was categorized as blood loss of < 20 mL/d (mild), 20 to 199 mL/d (moderate), and ≥ 200 mL/d (massive).

### Preprocedural CT Imaging

All BAE candidates at our institution underwent preprocedural contrast-enhanced multidetector CT (MDCT) imaging and at least one plain chest CT scan to identify the bleeding side and list the target vessels. MDCT imaging was performed using a 160- or 80-slice multidetector row CT scanner (Aquilion Precision or Aquilion PRIME; Canon Medical Systems). The arteries were enhanced by IV injection of a contrast material (iodine 300 or 370 mg/mL [Iopamiro, Bayer Yakuhin Ltd]; iodine 300 or 350 mg/mL [Omnipaque, GE Healthcare]) at a flow rate of 3 to 5 mL/s using trapezoidal cross-injection with an automated injector. The region of interest was the descending aorta. The scan was initiated when the CT value exceeded the threshold (250 Hounsfield units) during the arterial phase. Axial sections (1.0-mm thickness) were reconstructed at 0.25- or 0.5-mm intervals and exported for 3-dimensional simulation (Synapse Vincent; Fujifilm Holdings Corporation).

Imaging analysis was performed by 2 or 3 pulmonologists specializing in vascular interventions, with 9 to 15 years of experience in thoracic imaging and 4 to 12 years of experience in BAE. First, the potential bleeding sides were estimated according to the following: (1) findings suggestive of blood in the alveoli and airways (eg, ground-glass opacities, consolidations, and blood clots in the bronchial tree); or (2) the severity of underlying parenchymal abnormalities (eg, bronchiectasis and cavitary lesions). Potential culprit arteries were then listed according to the following criteria: (1) all bronchial arteries; (2) nonbronchial systemic arteries close to the potential bleeding lobe; and (3) nonbronchial systemic arteries larger than the contralateral vessel. Nonbronchial systemic arteries originating from the aorta include the intercostal artery, pulmonary ligament artery, and inferior phrenic artery.^11^ Nonbronchial systemic arteries originating from the subclavian/axillary artery include the internal thoracic artery, thyrocervical trunk, costocervical trunk, dorsal scapular artery, superior thoracic artery, thoracoacromial artery, lateral thoracic artery, and thoracodorsal artery.

### Bronchoscopy Prior to BAE

Bronchoscopy prior to BAE was performed at the discretion of the attending physician after CT examination to evaluate the site/side of bleeding, not for hemostasis. Bronchoscopy was performed without endotracheal intubation, and IV midazolam was used as a sedative. Additional studies, such as collection of airway secretions or bronchial lavage for culture/cytology, were also performed at the discretion of the attending physician. During the analysis, patients undergoing bronchoscopy were divided into 2 groups based on the interval between bronchoscopy and the last day of hemoptysis, and a 2-day cutoff was selected based on previous studies.^12^^,^^13^ Bronchoscopy-related complications were also recorded.

### Angiography and BAE

All angiography and BAE procedures were performed by 2 or 3 trained pulmonologists, each with 4 to 12 years of experience and who perform at least 50 BAE procedures per year. In our institution, pulmonologists perform all treatment procedures regarding hemoptysis (eg, medication, respiratory and critical care, bronchoscopy, interventional radiology). Angiographic procedures for aortic arteries were performed by using the femoral approach, whereas those for subclavian and axillary arteries were performed by using the radial or brachial approach. Aortography was not performed. All possible culprit arteries on MDCT imaging were then re-examined; angiography was performed by hand injection, as was selective angiography using a 4F or 5F guide catheter. Arteries with the following angiographic characteristics were considered abnormal and underwent super-selective embolization using a microcatheter: (1) densely stained lung parenchyma with hypervascularity (dilatation and tortuosity); (2) systemic-pulmonary shunting; or (3) aneurysmal formation.^8^^,^^11^^,^^14^ Platinum coils, N-butyl-2-cyanoacrylate, and gelatin sponge particles were used as embolic agents.

In the current study, embolization of unilateral/bilateral vessels was defined as that of unilateral/bilateral nonbronchial systemic arteries. Bronchial arteries were excluded from the definition because the common bronchial trunk is often present, which cannot be classified into left or right sides; in addition, bronchial arteries are prone to bilateral embolization because patients have relatively few bronchial arteries, and bronchial arteries are thought to have a higher risk of hemoptysis than other nonbronchial systemic arteries.^15^ Adverse events were classified based on the Society of Interventional Radiology Standards of Practice Committee guidelines.^16^

### Algorithm to Evaluate the Contribution of Bronchoscopy to BAE

**Figure 1:**
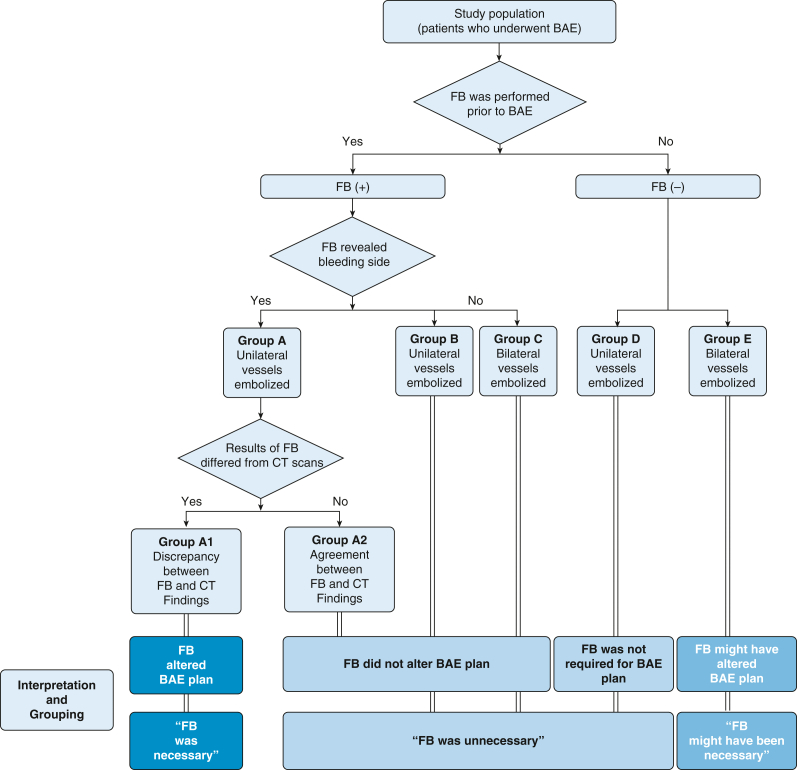
Method to evaluate the contribution of bronchoscopy to BAE. BAE = bronchial artery embolization; FB = flexible bronchoscopy.

The contribution of bronchoscopy in determining the side of embolization was evaluated as shown in Figure 1. First, patients who had undergone bronchoscopy were grouped into the following categories: bronchoscopy succeeded in lateralizing (and unilateral vessels were embolized) (Group A); bronchoscopy failed in lateralizing but unilateral vessels were embolized with reference to CT imaging and angiographic information (Group B); and bronchoscopy failed in lateralizing, and bilateral vessels were embolized (Group C).

Second, patients who had not undergone bronchoscopy were grouped into the following categories: unilateral vessels were embolized by using CT imaging and angiographic information (Group D), and the priority of the embolization side could not be decided and bilateral vessels were embolized (Group E).

Third, the preprocedural CT scans of patients in Group A were additionally read by 2 board-certified radiologists (H. S. and S. F.; 23 and 17 years of experience, respectively) who were blinded to the patients’ clinical information. The left/right predominance of the area of bronchiectasis and cavitary lesions was determined for each patient and categorized as “left dominant,” “right dominant,” or “unable to determine left-right difference.” Cases of disagreement were categorized as “unable to determine left-right difference.” Based on these additional categories, patients in Group A were further classified as patients whose categorization was “unable to determine left-right difference” or on opposite sides of the bronchoscopy results (Group A1) and patients whose categorization was on the same side as the bronchoscopy results (Group A2).

Finally, the number of patients in each group was counted and the contribution of bronchoscopy to BAE was determined. The percentage of patients in Group A1 indicated the percentage of patients for whom “bronchoscopy was necessary” (because bronchoscopy altered the BAE plan). The total percentage of patients in Groups A2, B, C, and D indicated the percentage of patients for whom “bronchoscopy was unnecessary” (because bronchoscopy did not alter the BAE plan or was not required for the BAE plan). The percentage of patients in Group E indicated the percentage of patients for whom “bronchoscopy might have been necessary” (because bronchoscopy might have altered the BAE plan).

### Data Analysis

Data collection was performed by retrospective analysis of medical records in November 2023 (90 days following the end of patient inclusion). The aforementioned additional CT assessments by 2 radiologists were performed in November 2023. The 90-day overall survival and hemoptysis-free survival rates following BAE were estimated by using the Kaplan-Meier method to verify the accuracy of the embolization plan. Events of concern for hemoptysis-free survival rate were defined as the recurrence of hemoptysis following BAE. Patients lost to follow-up were censored at the date of the last follow-up.

Analyses were performed by using R version 4.0.4 (R Foundation for Statistical Computing).

## Results

**Figure 2:**
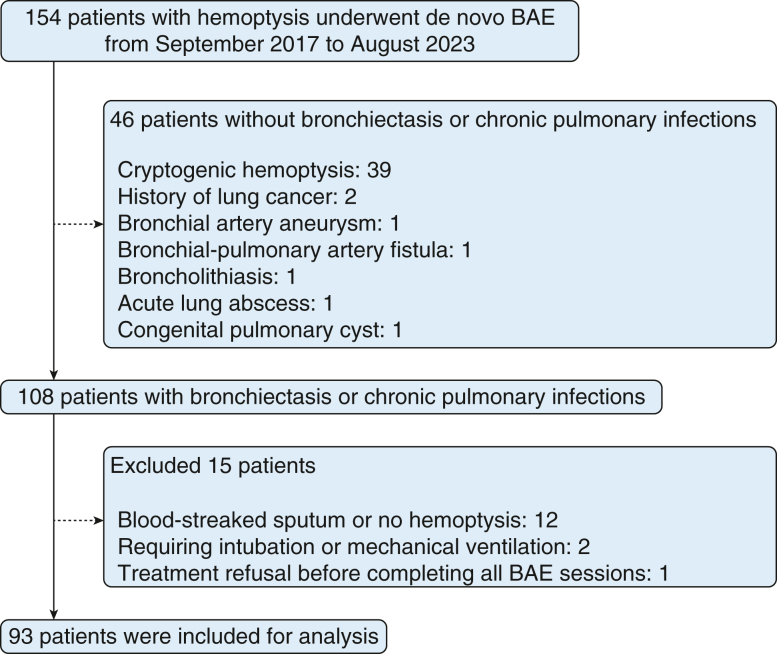
Flowchart of study inclusion. BAE = bronchial artery embolization.

Ninety-three patients (35 male patients and 58 female patients aged 27-90 years; median age, 74 years) were evaluated in this study (Fig 2). Characteristics of the patients are summarized in Table 1. The predominant etiologies were nontuberculous mycobacteriosis and non-cystic fibrosis bronchiectasis. The amount of hemoptysis was mild, moderate, and massive in 14 (15%), 60 (65%), and 19 (20%) patients, respectively. Eighty-two (88%) patients experienced recurrent episodes of hemoptysis. The median intervals between the last day of hemoptysis and the first CT scan, bronchoscopy, and BAE were 1 (interquartile range [IQR], 0-3; range, 0-191) day, 4 (IQR, 2-10.5; range, 1-34) days, and 15 (IQR, 9-28; range, 2-212) days.

**Table 1:** Characteristics of Study Patients (N = 93)

Characteristic	Value
Age, y	74 (64-78)
Sex	
Male	35 (38)
Female	58 (62)
Etiology	
NTM	37 (40)
Non-cystic fibrosis BE	25 (27)
History of TB	13 (14)
ASP	4 (4)
NTM with a history of TB	3 (3)
NTM with ASP	3 (3)
NTM with ASP and a history of TB	2 (2)
ASP with a history of TB	5 (5)
Active TB	1 (1)
Amount of bleeding	
Mild	14 (15)
Moderate	60 (65)
Massive	19 (20)
Recurrent episodes of hemoptysis	82 (88)
Examination before BAE	
CT imaging	93 (100)
FB	27 (29)
Days from last hemoptysis	
CT imaging	1 (0-3)
FB	4 (2-10.5)
BAE	15 (9-28)

Data are presented as medians and interquartile ranges (quartiles 1 to 3) or no. (%). ASP = aspergillosis; BAE = bronchial artery embolization; BE = bronchiectasis; FB = flexible bronchoscopy; NTM = nontuberculous mycobacteriosis.

The results of bronchoscopy are summarized in Table 2. Twenty-seven (29%) patients underwent bronchoscopy prior to BAE. The bleeding side was identified in 33% (9 of 27) of these patients. However, the side was identified in 70% (7 of 10) of patients undergoing bronchoscopy within 2 days of hemoptysis. Eight patients underwent observation only, and 19 patients underwent culture testing by collecting airway secretion or bronchial wash; 5 patients had previously unknown organisms detected (*Aspergillus* in 4 patients and *Mycobacterium avium* in 1 patient), and the remaining 22 patients had no new findings. No malignant tumor was found in any patient. Bronchoscopy-related complications occurred in 19% (5 of 27) of patients: exacerbation of bleeding (n = 2), hypoxemia due to laryngospasm requiring discontinuation of bronchoscopy (n = 1), paroxysmal supraventricular tachycardia (n = 1), and midazolam-induced disinhibition (n = 1). Among patients who underwent bronchoscopy within 2 days of hemoptysis, the rate of bronchoscopy-related complications was 30% (3 of 10).

**Table 2:** Results of Bronchoscopy Prior to BAE

	Interval Between Last Hemoptysis		Total (N = 27)
	≤ 2 Days (n = 10)	> 2 Days (n = 17)	
Age, y	75.5 (74.25-77)	68 (64-74)	74 (64-76)
No. of male patients	3 (30)	5 (29)	8 (30)
Examination			
Observation only	4 (40)	4 (24)	8 (30)
Bacteriologic tests^a^	6 (60)	13 (76)	19 (70)
Findings			
Identification of the side of bleeding	7 (70)	2 (12)	9 (33)
Detection of new bacteria	1^b^ (10)	4^c^ (24)	5 (19)
Detection of malignancy	0 (0)	0 (0)	0 (0)
Complications	3^d^ (30)	2^e^ (12)	5 (19)

Data are presented as medians and interquartile ranges (quartiles 1 to 3) or no. (%).

a Collection of airway secretion or bronchial wash.

b *Aspergillus* in a patient with nontuberculous mycobacteriosis (n = 1).

c *Mycobacterium avium* in patient with bronchiectasis (n = 1), *Aspergillus* in a patient with bronchiectasis (n = 1), *Aspergillus* in a patient with nontuberculous mycobacteriosis (n = 1), and *Aspergillus* in a patient with nontuberculous mycobacteriosis and history of TB (n = 1).

d Exacerbation of bleeding (n = 1), hypoxemia due to laryngospasm requiring discontinuation of bronchoscopy (n = 1), and paroxysmal supraventricular tachycardia (n = 1).

e Exacerbation of bleeding (n = 1), and disinhibition with midazolam (n = 1).

In total, 361 culprit arteries (152 bronchial and 209 nonbronchial systemic arteries) were identified. The median number of culprit arteries per patient was 3 (IQR, 2-5; range, 1-13), and 68 (73%) patients had nonbronchial systemic culprit arteries. Of the 361 culprit arteries, 351 (97%) were embolized; the remaining 10 arteries (4 bronchial and 6 nonbronchial systemic arteries) could not be embolized in whole or in part because they were difficult to select with the microcatheter because of narrowing or bending of vessels. A BAE-related adverse event requiring escalated level of care was observed in 1 (1%) patient who presented with incomplete paralysis of the left hand due to a postoperative hematoma from puncture of the left brachial artery.

Among 9 (10%) patients in whom bronchoscopy revealed the bleeding side (Group A), the additional CT assessment showed that bronchoscopy had provided information about the bleeding side that could not be determined by CT imaging alone in 1 (1%) patient (Group A1); the CT imaging in this patient showed no difference between the right and left side but not in the remaining 8 (9%) patients (Group A2). Among 18 (19%) patients in whom bronchoscopy did not reveal the bleeding side, unilateral and bilateral vessels were embolized in 13 (14%) (Group B) and 5 (5%) (Group C) patients, respectively.

**Figure 3:**
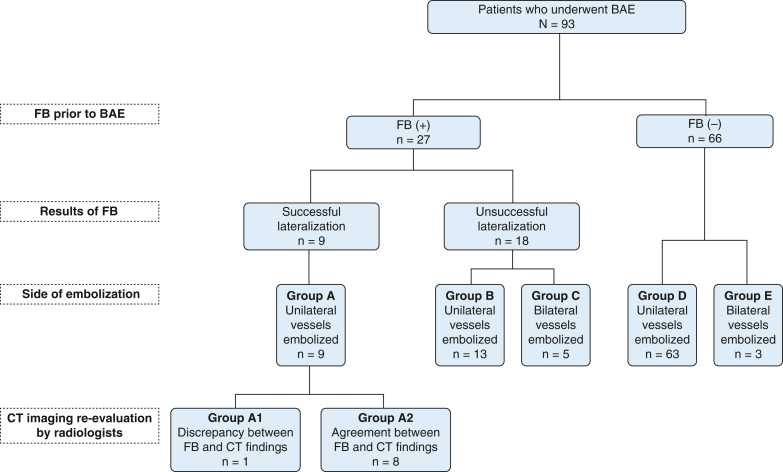
Flowchart of grouping according to the contribution of bronchoscopy to BAE. BAE = bronchial artery embolization; FB = flexible bronchoscopy.

Ten patients underwent bronchoscopy within 2 days of hemoptysis, including 1, 6, and 3 in Groups A1, A2, and B, respectively. Seventeen patients underwent bronchoscopy > 2 days after hemoptysis, including 2, 10, and 5 in Groups A2, B, and C. Bronchoscopy was not performed in 66 (71%) patients; unilateral and bilateral vessels were embolized in 63 (68%) patients (Group D) and 3 (3%) patients (Group E). “Bronchoscopy was necessary,” “bronchoscopy was unnecessary,” and “bronchoscopy might have been necessary” for 1 (1%), 89 (96%), and 3 (3%) patients (Fig 3).

**Figure 4:**
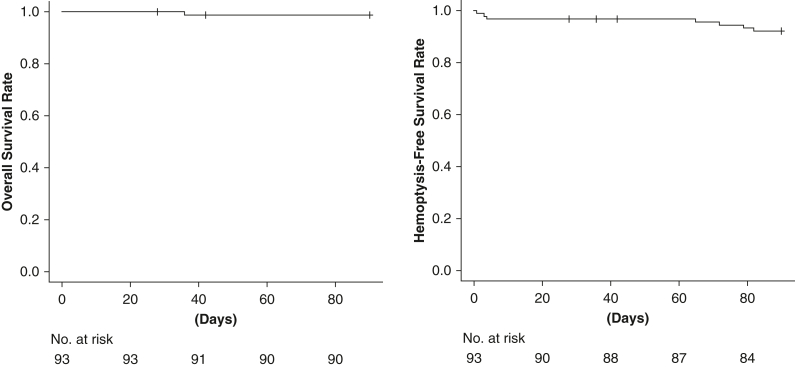
Kaplan-Meier estimate of overall survival (A) and hemoptysis-free survival (B).

The overall survival and hemoptysis-free survival rates at 90 days were 98.9% (95% CI, 92.5-99.8) and 92.3% (95% CI, 84.6-96.3), respectively (Fig 4). No patient died of hemoptysis during the 90-day postprocedural period; 1 patient died of pneumonia 36 days following the last BAE session. Seven patients experienced rebleeding during the 90-day postprocedural period, of whom 4 patients (1, 1, and 2 in Groups A, B, and D) had a successful lobectomy on the same side as embolization. The remaining 3 patients (1 in Group C and 2 in Group D) experienced resolution of bleeding using oral tranexamic acid; 2 patients survived > 5 years after BAE without additional intervention, and 1 patient died of pneumonia unrelated to hemoptysis 10 months following BAE.

## Discussion

This retrospective study showed that bronchoscopy contributed little to the planning of BAE in nonintubated patients with hemoptysis caused by bronchiectasis and chronic pulmonary infection. In our observations, 96% of patients did not require bronchoscopy for the planning of BAE. Importantly, the sides of hemorrhage identified by bronchoscopy were the same as those with the dominant lung involvement on CT imaging, suggesting that CT imaging alone is sufficient to identify the bleeding side prior to BAE. In addition, the accuracy of bronchoscopy to identify the bleeding side decreased over time, and bronchoscopy-related complications occurred in 19% (5 of 27) of cases.

In practice, flexible bronchoscopy is considered reliable for lateralization in patients with hemoptysis.^1^^,^^4^ However, in the current study, the bleeding side was determined in 68% (n = 63; Group D) of patients using only CT scanning and angiographic information. In addition, bronchoscopy failed to identify the bleeding side in 18 patients (Groups B and C); the side prioritized for embolization was determined by using CT imaging and angiographic information alone in the majority of cases (n = 13; Group B). Moreover, although the bleeding side was shown in bronchoscopy in 9 patients (Group A), it was also determined by CT alone in the majority of cases (n = 8; Group A2). Overall, 96% (89 of 93) of patients did not require bronchoscopy as part of their embolization plan. Furthermore, the overall survival and hemoptysis-free survival rates at 90 days were high, and no cases of treatment failure due to inappropriate choice of the side of embolization were observed. These results show that bronchoscopy actually contributed little to the planning of BAE and that CT imaging alone was sufficient to develop a treatment strategy for BAE. One possible factor could be the difference in the immediacy between these tools. In the current study, the median interval between hemoptysis and CT imaging and bronchoscopy was 1 and 4 days, respectively. Another possible factor is that not only hemorrhage but also underlying vascular abnormalities, knowledge of which is crucial for planning of BAE, are visualized on CT imaging but not on bronchoscopy.^17^

Flexible bronchoscopy has many roles in the overall management of hemoptysis, such as the diagnosis of etiologies, biopsy (especially in malignant cases), a guide for intubation, hemostatic procedure (eg, bronchial blocker placement and spigot), and clot extraction.^1^^,^^9^ However, based on our results, in patients whose etiologies are easy to diagnose and sources of bleeding are relatively easy to estimate from their pulmonary involvements observed via CT imaging (bronchiectasis and cavities), such as BAE candidates with bronchiectasis and chronic pulmonary infections, there is little need for bronchoscopy in the acute phase of hemoptysis as long as CT imaging is performed immediately following hemoptysis and BAE is scheduled for the near future. Several previous studies on massive hemoptysis or hemoptysis requiring ICU treatment, although they were small studies with heterogeneous populations, also reported that radiographic assessment could replace bronchoscopy for determining the site/side of bleeding.^7^^,^^12^ Bronchoscopy prior to BAE seems to have become less critical owing to the routine use of MDCT imaging since the 2000s,^6^^,^^7^^,^^18^ unlike in the past when CT imaging and bronchoscopy were complementary in the localization and diagnosis of hemoptysis.^19^

The current results also suggest that bronchoscopy should be conducted promptly if CT imaging fails to identify the side of bleeding. In this study, the bleeding detected by bronchoscopy increased from 33% (9 of 27) to 70% (7 of 10) when the procedure was performed within 2 days of hemoptysis. This finding is consistent with that of previous studies on massive hemoptysis,^7^^,^^12^ which reported that the source of active bleeding is most likely to be localized when bronchoscopy is performed during active hemoptysis or within 24 to 48 hours of cessation. Conversely, a multicenter study reported that early bronchoscopy (ie, during active bleeding or ≤ 48 hours after hemoptysis ceased) in detecting the source of bleeding was only beneficial for cases with a high volume of hemoptysis (>20 mL/d)^13^; therefore, selecting cases with massive hemoptysis may increase the diagnostic rate of bronchoscopy.

The complications of bronchoscopy, which occurred in 19% (5 of 27) of cases, were also highlighted in the current study. Moreover, the complication rate increased to 30% (3 of 10) when the procedure was performed within 2 days of hemoptysis. Complications leading to respiratory compromise are of greater concern for nonintubated patients with hemoptysis in general wards; for intubated patients, these complications are manageable because bronchoscopy is easy and safe to perform via the endotracheal tube and high concentration oxygen can be administered quickly. Special attention should be paid to patients with bronchiectasis and chronic lung infections, as their respiratory function tends to be compromised owing to lung inflammation and deformities caused by bronchiectasis and cavities.

Overall, the current study findings do not support routine bronchoscopy prior to BAE in nonintubated patients with hemoptysis caused by bronchiectasis and chronic pulmonary infection. Bronchoscopy may be considered in patients whose bleeding side is difficult to determine using CT imaging due to equal severity of lung involvement in both lungs within 2 days after hemoptysis, with caution taken for respiratory status. As for bacteriologic evaluation, previously unknown organisms were detected in 5 of the 19 patients undergoing bronchoscopic sputum culture; however, such evaluations can be performed postoperatively when patients are more stable because they do not affect the acute hemostasis strategy.

The current study has some limitations. First, this was a retrospective and single-center study. Second, the indication and timing of bronchoscopy were not uniform. The decision on whether to perform a bronchoscopy was based on several factors, including the judgment of the attending physician, the physical status of the patient, and the patient’s request. The utility of bronchoscopy might have been underestimated because the time from hemoptysis to bronchoscopy was longer than that of CT imaging. However, even if all 5 patients in Group C (those who underwent bronchoscopy > 2 days following hemoptysis but both bronchoscopy and CT imaging failed in lateralizing) had undergone earlier and successful bronchoscopy, the percentage of patients who did not require bronchoscopy for the planning of BAE would have been < 90% (84 of 93). In addition, a prospective study on this issue would be difficult to conduct because it is less feasible and ethical due to the risk of complications, as reported in the current study (19%). Third, this study did not determine the benefits or harms of bronchoscopy prior to BAE in the setting of other leading causes of hemoptysis such as lung cancer and cryptogenic hemoptysis. The conclusion of this study cannot be applied to such cases; bronchoscopic biopsy (histologic evaluation) is of relatively high importance in patients with lung cancer, especially when making decisions about chemotherapy, and bronchoscopy is generally required to rule out other causes of bleeding in patients with cryptogenic hemoptysis.

## Interpretation

This study showed that bronchoscopy contributed little to the planning of BAE in nonintubated patients with hemoptysis caused by bronchiectasis and chronic pulmonary infection. Our findings do not support the routine use of bronchoscopy prior to BAE in this population. Bronchoscopy may be considered in patients whose side of bleeding is difficult to determine using CT imaging due to equal severity of lung involvement in both lungs within 2 days after hemoptysis, with caution taken for respiratory status.

## Funding

The authors have reported to *CHEST* that no funding was received for this study.

## Financial/Nonfinancial Disclosures

The authors have reported to *CHEST* the following: H. I. discloses no relevant relationships related to the current article. Activities by H. I. not related to the current article including the following: institution has grants/grants pending from Terumo; and payment for lectures, including service on speakers bureaus from Boston Scientific Japan, Terumo, Piolax Medical Devices, and Stryker Japan. Other relationships: disclosed no relevant relationships. None declared (T. N., K. T., S. F., H. S.).
